# Prevalence of the Wish to be of the Opposite Gender in Adolescents and Adults with Autism Spectrum Disorder

**DOI:** 10.1007/s10508-018-1218-3

**Published:** 2018-05-07

**Authors:** Anna I. R. van der Miesen, Hannah Hurley, Anneloes M. Bal, Annelou L. C. de Vries

**Affiliations:** 1 Department of Child and Adolescent Psychiatry, Center of Expertise on Gender Dysphoria, VU University Medical Center, PO Box 7057, 1007 MB Amsterdam, The Netherlands; 2 Dr. Leo Kannerhuis, Center for Autism, Amsterdam, The Netherlands

**Keywords:** Autism spectrum disorder, Gender dysphoria, Gender identity disorder, Gender variance

## Abstract

Several studies have suggested an overrepresentation of (symptoms of) autism spectrum disorder (ASD) among individuals with gender dysphoria. Three studies have taken the inverse approach in children with ASD and showed increased parent report of the wish to be of the opposite gender in this group. This study compared the self-reported wish to be of the opposite gender (one item of the Youth Self-Report [YSR] and the Adult Self-Report [ASR]) of 573 adolescents (469 assigned boys and 104 assigned girls) and 807 adults (616 assigned males and 191 assigned females) with ASD to 1016 adolescents and 846 adults from the general population. Emotional and behavioral problems were measured by the DSM-oriented scales of the YSR and ASR. In addition, the Children’s Social Behavior Questionnaire and the Adult Social Behavior Questionnaire were used to measure specific subdomains of the ASD spectrum to test whether specific subdomains of ASD were particularly involved. Significantly more adolescents (6.5%) and adults (11.4%) with ASD endorsed this item as compared to the general population (3–5%). In adolescents, assigned girls endorsed this item more than assigned boys. No significant gender differences were found in the adults with ASD. In addition, on all DSM-oriented scales of both the YSR and ASR, adolescents and adults with ASD who endorsed the gender item had significantly higher scores compared to those without. There were no significant associations between endorsement of the gender item and any specific subdomain of ASD, providing no evidence for a sole role of one of the ASD subdomains and endorsement of the wish to be the opposite gender.

## Introduction

The co-occurrence of gender dysphoria (GD) and autism spectrum disorder (ASD) is gaining increased clinical and research interest (van der Miesen, Hurley, & de Vries, 2016). As stated in the *Diagnostic and Statistical Manual of Mental Disorders* (DSM-5), the core criterion of GD is a marked incongruence between one’s experienced and one’s assigned gender (American Psychiatric Association, 2013).^1^ ASD is characterized by problems in social interaction and communication along with repetitive behavior and specific interests (APA, 2013). Examples of communication problems consist of having difficulties of understanding the point of view of others, problems with making eye contact, and no sharing or inadequate sharing of joy in interests. Examples of repetitive behavior and specific interests include intense interests in hobbies, activities or topics, repeating behaviors and getting upset by a slight change in routines (APA, 2013).

Several case reports have described the co-occurrence of GD and ASD (Gallucci, Hackerman, & Schmidt, 2005; Jacobs, Rachlin, Erickson-Schroth, & Janssen, 2014; Kraemer, Delsignore, Gundelfinger, Schnyder, & Hepp, 2005; Landen & Rasmussen, 1997; Lemaire, Thomazeau, & Bonnet-Brilhault, 2014; Mukaddes, 2002; Parkinson, 2014; Perera, Gadambanathan, & Weerasiri, 2003; Tateno, Tateno, & Saito, 2008; Tateno, Teo, & Tateno, 2015; Williams, Allard, & Sears, 1996). Using the Dutch version of the Diagnostic Interview for Social and Communication Disorders-10th revision (DISCO-10; Wing, 1999) in a group of children and adolescents with GD, there was an overrepresentation of ASD diagnoses with a prevalence of 7.8% (de Vries, Noens, Cohen-Kettenis, van Berckelaer-Onnes, & Doreleijers, 2010). In addition, one study of children and adolescents consecutively referred to a gender identity service found an ASD diagnosis in 2.3% and pervasive developmental disorder in 9.3% (Spack et al., 2012). Other studies focused on symptoms of ASD in adults with GD (Jones et al., 2012; Pasterski, Gilligan, & Curtis, 2014). Using the self-report Autism Spectrum Quotient (AQ), Jones et al. found an increased number of symptoms of ASD in adults with GD compared to non-referred adults. Pasterski et al., using the same measure, also reported an increased ASD trait rate in adults with GD compared to non-referred adults (for an overview of the literature on GD and ASD, see George & Stokes, 2016; van der Miesen et al., 2016).

To date, three studies investigated symptoms of ASD in children and adolescents with GD. First, Skagerberg, Di Ceglie, and Carmichael (2015) found increased symptom levels of ASD compared to a non-referred sample using the Social Responsiveness Scale (SRS; Constantino & Gruber, 2005). Second, a study by Shumer, Reisner, Edwards-Leeper, and Tishelman (2016) found that 23.1% of individuals presenting at a multidisciplinary gender clinic had possible, likely, or very likely Asperger’s disorder. Third, a study of ASD symptomatology in children and adolescents with GD by van der Miesen, de Vries, Steensma, and Hartman (2018) investigated different subdomains of the autism spectrum using the Children’s Social Behavior Questionnaire (CSBQ; Hartman, Luteijn, Moorlag, de Bildt, & Minderaa, 2015). It was demonstrated not only that children and adolescents with GD had a higher mean score on the total CSBQ than non-referred children and adolescents, but also scored higher on all the specific subdomains of the CSBQ: tuned behavior, social interaction, orientation, understanding, stereotyped behavior, and resistance to change, indicating that more than one subdomain of the ASD spectrum might be involved in the co-occurrence of GD and (symptoms of) ASD contrary to what has been suggested in the literature (for an overview of the suggested hypotheses, see van der Miesen et al., 2016).

As such, findings suggest a potential co-occurrence of GD and ASD (symptoms), but all these previous studies were conducted with individuals referred to specialized gender identity clinics. So far, only three studies have examined symptoms of GD, defined as gender variance, in a population with ASD (Janssen, Huang, & Duncan, 2016; May, Pang, & Williams, 2017; Strang et al., 2014). In the study of Strang et al., children diagnosed with ASD and children diagnosed with other neurodevelopmental disorders (e.g., attention deficit hyperactivity disorder; ADHD) were investigated. Gender variance was defined as the wish to be of the opposite gender as measured by one item (Item 110) of the Child Behavior Checklist (CBCL; Achenbach & Rescorla, 2001).^2^ In the past, two specialized gender identity services studied the endorsement of Item 110 of the CBCL and found strong correlations between a clinical GD diagnosis and endorsement of this item (Cohen-Kettenis, Owen, Kaijser, Bradley, & Zucker, 2003). In addition, they reported that the item-level data were sensitive and specific to those children with a clinical diagnosis of GD. Although a wish to be of the opposite gender is not necessarily related to a GD diagnosis according to the DSM or to the broader concept of gender nonconformity, these strong correlations suggest that this item is a valid measure for this aspect of GD. Strang et al. found that parents of 5.4% of the 147 probands of children with ASD reported that their child sometimes or frequently wished to be of the opposite gender. This was 7.59 times more likely than in children from the non-referred comparison group. No significant differences were found between assigned boys and assigned girls with ASD. Another important finding of Strang et al. was that the children with both neurodevelopmental disorders (ASD and ADHD) and endorsement of this item showed significantly more emotional symptoms reported on the CBCL than children with ASD only, but comparing the non-ASD neurodevelopmental groups among participants with ASD alone, no increased emotional symptoms were found. The first finding is in concordance with other reports that demonstrated more anxiety and mood disorders in children and adolescents with GD compared to general population samples (e.g., de Vries, Doreleijers, Steensma, & Cohen-Kettenis, 2011).

Janssen et al. (2016) and May et al. (2017) took the same approach in children with ASD. Janssen et al. found that 5.1% of the parents endorsed Item 110 of the CBCL, while May et al. found this for 4%, with both studies finding no significant differences between assigned boys and girls at birth.

Since these three studies focused on this gender item in children with ASD, it is uncertain whether increased endorsement of this item would appear in adults with ASD as well, and whether these findings in children with ASD could be replicated using a self-report questionnaire instead of a parent-report questionnaire. Therefore, in the present study, we investigated endorsement of the wish to be of the opposite gender in both an adolescent and adult population with ASD using a self-report questionnaire.

Confirmation of the increased prevalence of the wish to be of the opposite gender in an adolescent population with ASD and the first finding of an increased wish to be of the opposite gender in an adult population with ASD would further elucidate the existence of endorsement of this item in individuals with ASD and emphasize the need for clinical guidelines. Four hypotheses were examined in the current study. First, in agreement with the studies in children with ASD (Janssen et al., 2016; May et al., 2017; Strang et al., 2014), a higher prevalence of the wish to be of the opposite gender was expected in the adolescents with ASD. We expected to find a higher prevalence of endorsement of this item in adults with ASD as well, as previous studies suggested that symptoms of ASD are increased in adults with GD (Jones et al., 2012; Pasterski et al., 2014). Second, after accounting for the underlying uneven sex ratio in ASD (preponderance of males), we expected to find relatively equal numbers of assigned males and females with ASD endorsing the gender item, in agreement with the studies conducted in children with ASD (Janssen et al., 2016; Strang et al., 2014). Third, we hypothesized that all specific subdomains of autism would be more prevalent in individuals with a co-occurring wish to be of the opposite gender as one prior study found that all subdomains of autism spectrum symptoms, as measured by the CSBQ, were increased in a child and adolescent population with GD (van der Miesen et al., 2018). In agreement with these findings, we predicted to find not only similar rates of symptoms on different subdomains of ASD in adolescents, but also in adults with and without an increased prevalence of the wish to be of the opposite gender. Fourth, we expected increased emotional (anxious and depressive) problems in adolescents and adults with both ASD and endorsement of the gender item, compared to adolescents and adults with ASD but without this endorsement given the evidence for increased emotional (anxiety and depressive) symptoms in children reported in the literature (de Vries et al., 2011). Also, there is evidence for more psychiatric problems (mainly affective and anxiety disorders) in adults with GD compared to general population samples (e.g., Heylens et al., 2014; Zucker, Lawrence, & Kreukels, 2016).

## Method

### Participants and Procedure

A total of 3242 individuals participated in the study: A sample of Dutch individuals diagnosed with ASD (573 adolescents and 807 adults) were compared to 1016 non-referred adolescents from a Dutch standardization sample of the Youth Self-Report (YSR) and 846 non-referred adults from a U.S. national standardization sample of the Adult Self-Report (ASR) as there is no Dutch adult standardization sample (Achenbach & Rescorla, 2003; Verhulst, van der Ende, & Koot, 1997). Between March 2010 and October 2014, individuals with ASD who were referred to the “Dr. Leo Kannerhuis (LKH)” for treatment of ASD participated in this study. The “LKH” is a tertiary mental health clinic specialized in the treatment of ASD with several locations throughout the Netherlands. At their referral to the clinic, all clients were diagnosed by a clinician according to the DSM-IV diagnostic criteria (APA, 2000). Only individuals with both Verbal and Performance IQ scores higher than 70 were included. Clients referred by the Center of Expertise on Gender Dysphoria of the VU University Medical Center (VUmc) in Amsterdam were excluded (*n* = 2).

At the start of treatment, all clients completed several self-report questionnaires as part of the Routine Outcome Monitoring (ROM), a method devised to systematically collect data on the effectiveness of mental health clinics and monitoring client outcomes (de Beurs et al., 2011). The ROM that is used at the “LKH” consists of two layers of self-report questionnaires. The first layer is a set of questionnaires that is used in all mental health clinics in the Netherlands measuring behavioral and emotional functioning, and the second layer consists of several self-report questionnaires regarding symptoms of ASD (Bal, Cuppen, & Teunisse, 2013). The Medical Ethical Committee of the “LKH” approved the study, and all participants and parents in this study gave written informed consent for the use of these questionnaires in scientific publications.

#### Adolescents

The study sample consisted of 573 adolescents with ASD (*M* age = 15.98 years, SD = 1.85), including 104 girls (*M* age = 16.13 years, SD = 1.66) and 469 boys (*M* age = 15.95 years, SD = 1.89). A chi-square test of independence showed that significantly more assigned boys with ASD were included than assigned girls, *χ*^2^(1) = 150.64, *p* < .001. The adolescents with ASD were compared to the YSR standardization sample, which consisted of 1016 non-referred adolescents (521 girls and 495 boys; Verhulst et al., 1997). This standardization sample was drawn from a Dutch national sample of non-referred adolescents (11–18 years old), who had not received mental health care or special education within the past 12 months or were not referred for mental health care because of emotional and behavioral problems (Verhulst et al., 1997).

#### Adults

A total group of 807 adults with ASD (*M* age = 32.14 years, SD = 12.86), 191 assigned females (*M* age = 30.01 years, SD = 11.24) and 616 assigned males (*M* age = 32.74 years, SD = 13.24), participated in the current study. A chi-square test of independence showed that significantly more assigned males with ASD were included than females, *χ*^2^(1) = 203.18, *p* < .001. The U.S. national standardization sample of the ASR was used as a comparison group, which consisted of 846 non-referred adults, 465 females and 381 males (*M* age = 29.9 years, SD = 9.5; Achenbach & Rescorla, 2003). Non-referred adults had not received mental health services or attended special school classes within the past 12 months (Achenbach & Rescorla, 2003).

### Measures

#### Adolescents

##### The Wish to be of the Opposite Gender

Item 110 (I wish to be of the opposite sex) of the YSR, a standardized 118-item self-report questionnaire of behavioral and emotional functioning for ages 11–18, was used to measure gender incongruence (Verhulst et al., 1997). On the YSR, adolescents can report whether they have experienced the item “never,” “sometimes,” or “often” in the past 6 months. As in previous studies, responses for Item 110 were dichotomized into two groups: those with no wish to be of the opposite gender (“never”) and those who “sometimes” or “often” wished to be of the opposite gender (Strang et al., 2014).

##### DSM-Oriented Problems

The DSM-oriented problem scales (affective problems, anxiety problems, somatic problems, attention deficit/hyperactivity problems, conduct problems, and oppositional defiant problems) of the YSR were used to measure internalizing and externalizing problems (Verhulst et al., 1997). The adolescents were asked to rate the frequency of each behavior over the previous 6 months on a three-point Likert scale (0 = not true, 1 = somewhat or sometimes true, 2 = very true or often true). The DSM-oriented scales are displayed on profiles that indicate *T*-scores comparing the adolescent’s scores with scores from the national normative sample of the adolescent’s gender and age. Standardized *T*-scores are presented (*M* = 50, SD = 10), with cutoffs for the borderline clinical range (64 < *T* < 70) and clinical range (*T* > 70). Gender-, age-, and informant-specific *T*-score cutoff points were based on Dutch national normative samples of adolescents who had not received mental health services or special education services in the preceding 12 months (Verhulst et al., 1997). The criterion and construct validity of the YSR are adequate (Evers, van Vliet-Mulder, & Groot, 2006).

##### Autistic Symptoms

The Children’s Social Behavior Questionnaire (CSBQ) is a 49-item questionnaire that was used to measure the various behavioral and emotional problems as they are seen in ASD (Hartman, Luteijn, Serra, & Minderaa, 2006; Hartman et al., 2015). The items were rated on a three-point scale (0 = not applicable, 1 = sometimes or a little applicable, 2 = very applicable) by the parents or caregivers. The CSBQ consists of six subscales: (1) behavior not optimally tuned to the social situation (not tuned), (2) reduced contact and social interest (social), (3) difficulties in understanding social information (understanding), (4) orientation problems in time, place, or activity (orientation), (5) stereotyped behaviors (stereotyped), and (6) fear of and resistance to change (change). In a large Dutch sample study, the CSBQ was found to be a reliable and valid instrument (Hartman et al., 2006).

#### Adults

##### The Wish to be of the Opposite Gender

The wish to be of the opposite gender was measured by Item 110 (I wish to be of the opposite sex) of the ASR, a standardized self-report questionnaire of emotional and behavioral functioning for ages 18–59 (Achenbach & Rescorla, 2003). On the ASR, individuals can report whether they have experienced the item “never,” “sometimes,” or “often” over the past 6 months. Possible responses on the ASR were dichotomized into two groups: those with no wish to be of the opposite gender (“never”) and those who “sometimes” or “often” wished to be of the opposite gender.

##### DSM-Oriented Problems

The DSM-oriented problem scales (depressive problems, anxiety problems, somatic problems, avoidant personality problems, attention deficit/hyperactivity problems, and antisocial personality problems) were drawn from 126 items of the ASR (Achenbach & Rescorla, 2003). The items assess emotional and behavioral problems over the previous 6 months, with three response options (0 = not true, 1 = somewhat or sometimes true, 2 = very true or often true). Standardized *T*-scores of 70 and above are generally considered clinically significant and scores between 65 and 70 identify the borderline clinical range. The criterion and construct validity of the ASR are high (Achenbach & Rescorla, 2003).

##### Autistic Symptoms

The Adult Social Behavior Questionnaire (ASBQ), a self-report questionnaire, was used to measure distinct problem domains within ASD. The ASBQ is an adaptation of the CSBQ (van den Bosch & Minderaa, 2002). The 150 items are reported by the clients on a three-point Likert scale: “not applicable,” “sometimes or a little applicable,” or “very applicable.” The items can be summed into four subscales: (1) qualitative impairment in social interaction, (2) qualitative impairment in social communication, (3) limited, repetitive, stereotyped patterns, and (4) associated features. The reliability and validity of the ASBQ are good (Horwitz et al., 2016).

### Statistical Analyses

Data were analyzed using SPSS version 22.0. A significance level of *p* < .05 (two-tailed) was used. Data were checked for missing values and outliers, and normality assumptions were tested. First, percentages of endorsement of Item 110 among groups were calculated and relative risk odds ratio (OR) analyses were applied (Fleiss, 1981). The statistical significance of the OR was evaluated by computing confidence intervals and *p* values (Bland & Altman, 1999). Sex ratio differences were calculated using chi-square tests. Second, independent samples *t* tests and additional multivariate profile analysis were employed to evaluate differences in mean DSM-oriented *T*-scores between participants with ASD for Item 110 status (with or without endorsement) and the non-referred group. The DSM-oriented *T*-scores of adolescents were compared with *T*-scores of adolescents in a Dutch standardization sample, and for adults the same comparison for *T*-scores was used with a U.S. national standardization sample. Third, a set of Pearson correlations were used to examine the relationship between symptoms of ASD and the gender item for adolescents and adults, respectively. Finally, Cohen’s *d* was used to calculate effect sizes between the groups with and without reported endorsement of Item 110. Effect sizes of 0.80 or more were considered as large, effect sizes of 0.50–0.79 were considered as medium, 0.20–0.49 as small, and less than 0.20 as negligible (Cohen, 1988).

## Results

### Adolescents

The prevalence of self-reported endorsement of Item 110 in the 573 adolescents with ASD was 6.5% (*n* = 37 [*n* = 31 “sometimes” and *n* = 6 “often”]) and 3.1% (*n* = 32) in the non-referred controls. As compared to the YSR non-referred controls, adolescents with ASD were 2.12 times more likely to show endorsement of this item (*p *= .002, 95% CI 1.31–3.45). The prevalence was 5.3% (*n* = 25) in assigned boys and 11.5% (*n* = 12) in assigned girls with ASD. In the non-referred controls, boys showed a prevalence of endorsement of Item 110 of 2.0% (*n* = 10) and girls of 4.2% (*n* = 22). Odds ratios showed that boys with ASD were 2.73 times more likely to show endorsement of Item 110 (*p* = .008, CI 1.30–5.75) and girls with ASD were 2.96 times more likely to endorse this item (*p* = .004, CI 1.41–6.19).

The mean age of adolescents with endorsement of this item (*M* age = 16.13 years, SE = 1.74) was not significantly different from those without (*M* age = 15.98 years, SE = 1.86, *t *= − 0.47, *df* = 572, *p* = .640).

After accounting for sex ratio differences by randomly removing subjects to achieve a similar ratio, and repeating this technique three times in the YSR standardization sample, significant differences in sex ratios between the ASD sample with endorsement of Item 110 and the non-referred controls with endorsement of this item were found in favor of girls (*p* = .03).

Adolescents with endorsement of Item 110 and ASD had significantly higher DSM-oriented *T*-scores on the YSR as compared to adolescents with ASD but without endorsement of the item (Table 1). Additional multivariate profile analyses confirmed the main effect for group. Adolescents with endorsement of Item 110 and ASD had significantly higher DSM-oriented *T*-scores compared to adolescents with ASD but without endorsement of the item (*p* < .001). No significant differences were found between those who endorsed this item sometimes versus often. In addition, an interaction effect was found. Subsequent univariate analyses indicated that the group-by-gender interaction effects were present on the subscales oppositional defiant problems and conduct problems. A within-group post hoc *t* test revealed that on the oppositional defiant problems scale the interaction arose because in the sample without endorsement of Item 110, assigned boys had a somewhat lower mean score than assigned girls (see Table 1 for mean scores for assigned boys and girls at birth separately), but in the group with endorsement of Item 110, the assigned boys scored significantly higher than the girls. On the conduct problems scale, the interaction arose because in the sample without endorsement of Item 110 assigned boys and girls did not differ on these scales, while in the group with endorsement of Item 110 the assigned boys scored significantly higher than girls.

**Table 1:** Comparison of DSM-oriented total group *T*-scores^a^ in adolescents with ASD and with or without endorsement of Item 110

	Group												*t* ^b^	*df*	*p*	*d*
	ASD without endorsement of Item 110						ASD with endorsement of Item 110									
	Assigned boys (*n* = 444)		Assigned girls (*n* = 92)		Total (*n* = 536)		Assigned boys (*n* = 25)		Assigned girls (*n* = 12)		Total (*n* = 37)					
	*M*	SD	*M*	SD	*M*	SD	*M*	SD	*M*	SD	*M*	SD				
Affective problems	58.86	8.32	60.08	7.96	59.07	8.26	68.72	9.36	66.42	6.58	67.97	8.53	− 6.33	571	<.001	1.08
Anxiety problems	56.97	7.43	59.77	8.70	57.45	7.73	64.64	6.35	64.33	8.02	64.54	6.82	− 5.44	571	<.001	0.92
Somatic problems	56,43	7.91	57.30	7.90	56.58	7.91	62.68	9.39	59.08	5.07	61.51	8.34	− 3.65	571	<.001	0.62
Attention deficit/hyperactivity problems	57.68	7.21	58.55	7.33	57.82	7.23	61.76	7.60	60.33	5.94	61.30	7.05	− 2.83	571	.005	0.48
Oppositional defiant problems	54.36	5.46	54.60	5.17	54.40	5.41	58.32	6.99	54.00	5.10	56.92	6.68	− 2.24	39.32	.031	0.46
Conduct problems	55.58	6.31	55.67	6.47	55.60	6.33	61.52	7.62	55.17	6.83	59.46	7.88	− 2.92	39.28	.006	0.60

^a^Absolute range for total Youth Self-Report *T*-scores is 50–94

^b^*t* tests were conducted comparing the total groups

There were no significant correlations between endorsement of Item 110 and specific subdomains of the ASD spectrum. In general, endorsement of Item 110 was not associated with specific subdomains of ASD.

### Adults

The prevalence of endorsement of Item 110 in adults with ASD was 11.4% (*n* = 92 [*n *=* 75* “sometimes” and *n* = 17 “often”]), which is 2.46 times more likely than the 5.0% (*n* = 42) prevalence of the gender item in the non-referred adults of the ASR standardization sample (*p* < .001, CI 1.69–3.60). The prevalence of endorsement of Item 110 in assigned females with ASD was 15.8% (*n *= 30) and 10.0% (*n* = 62) in assigned males with ASD. In the non-referred adults, the prevalence of this item in females was 6.0% and in males 2.9%. Odds ratios for the wish to be of the opposite gender in adults showed that males with ASD were 3.76 times more likely to endorse Item 110 (*p* < .001, CI 1.96–7.24) and females with ASD were 3.02 times more likely to endorse this item (*p* < .001, CI 1.74–5.24).

The mean age of adults with endorsement of Item 110 (*M* age = 33.73 years, SE = 13.58) was not significantly different from those without (*M* age = 31.30 years, SE = 12.59, *t *= − 1.7, *df* = 801, *p* = .080).

Sex ratios of participants with endorsement of Item 110 and ASD were not significantly different from the sex ratios of the ASR standardization sample with endorsement of this item after accounting for sex ratio differences in the ASR standardization sample.

Adults with endorsement of Item 110 and ASD had significantly higher DSM-oriented *T*-scores on the ASR as compared to adults with ASD but without endorsement of this item (Table 2). Additional multivariate profile analyses confirmed the main effect for group. Adults with endorsement of Item 110 and ASD had significantly higher DSM-oriented *T*-scores compared to adults with ASD but without endorsement of the item (*p* < .001). No significant interaction effect for gender was found. No significant differences were found between those who endorsed this item sometimes versus often.

**Table 2:** Comparison of DSM-oriented total group *T*-scores^a^ in adults with ASD with and without endorsement of Item 110

	Group												*t* ^b^	*df*	*p*	*d*
	ASD without endorsement of Item 110						ASD with endorsement of Item 110									
	Assigned males (*n* = 554)		Assigned females (*n* = 161)		Total (*n* = 715)		Assigned males (*n* = 62)		Assigned females (*n* = 30)		Total (*n* = 92)					
	*M*	SD	*M*	SD	*M*	SD	*M*	SD	*M*	SD	*M*	SD				
Depressive problems	63.83	10.53	69.02	10.76	65.00	10.79	71.26	9.89	72.67	10.25	71.51	10.08	− 5.66	805	<.001	0.61
Anxiety problems	57.65	7.72	62.75	8.58	58.80	8.21	62.13	8.30	64.73	8.28	62.82	8.31	− 4.69	805	<.001	0.49
Somatic problems	57.42	7.95	61.44	9.59	58.32	8.50	60.45	7.69	64.50	10.37	61.72	8.84	− .3.64	805	<.001	0.40
Avoidant personality problems	66.05	10.63	69.86	9.72	66.94	10.53	70.84	10.75	71.77	10.64	70.74	10.89	− 3.62	805	<.001	0.36
Attention deficit/hyperactivity problems	60.38	7.93	62.53	9.94	60.87	8.46	62.87	7.30	67.07	9.84	64.06	8.52	− 3.60	805	<.001	0.38
Antisocial personality problems	56.29	7.00	56.14	6.03	56.25	6.79	59.31	7.81	60.63	8.52	59.57	7.98	− 3.99	108.40	<.001	0.48

^a^Absolute range for total Adult Self-Report *T*-scores is 50–96

^b^*t* tests were conducted comparing the total groups

We found correlations between endorsement of the gender item and all ASD subdomains, consisting of qualitative impairment in social interaction (*r* = .11, *n *= 716, *p* = .010), qualitative impairment in social communication (*r* = .12, *n* = 716, *p* = .010), limited, repetitive, stereotyped patterns (*r* = .15, *n* = 716, *p* < .001), and associated features (*r* = .14, *n* = 716, *p* < .001). Further independent *t* tests revealed that, on all subscales, participants with ASD and co-occurring endorsement of Item 110 scored significantly higher on all ASD symptoms than those participants with ASD without (*p* < .05).

## Discussion

As hypothesized, the present study not only confirmed an earlier finding of increased endorsement of Item 110 in children with ASD (Janssen et al., 2016; May et al., 2017; Strang et al., 2014), but is also the first study to find the same results in adults with ASD. Whereas parent-reported endorsement of this item was 7.59 times more likely in the Strang et al. study and 7.76 times more likely in the Janssen et al. study, this time self-reported endorsement of Item 110 was 2.12 times more likely in adolescents and 2.46 times more likely in adults with ASD compared to non-referred standardization samples of the YSR and ASR, respectively (Achenbach & Rescorla, 2003; Verhulst et al., 1997). The findings were in line with the previously reported overrepresentation of ASD in individuals with GD (de Vries et al., 2010; Edwards-Leeper & Spack, 2012; Jones et al., 2012; Pasterski et al., 2014; Shumer et al., 2016; Skagerberg et al., 2015) and provide evidence of an increased wish to be of the opposite gender in adults with ASD. It should be realized that endorsement of this item measured with the YSR/ASR does not imply a GD diagnosis. In addition, more individuals stated that they “sometimes” rather than “often” wished to be of the opposite gender. Further, this endorsement occurred in only 6.5% of the adolescents and in 11.4% of the adults. Despite these facts, the increased prevalence of wish to be of the opposite gender in an ASD population compared to the general population deserves further study.

Contrary to our second hypothesis, adolescent girls with ASD had a significantly higher prevalence of endorsement of Item 110 compared to adolescent boys with ASD. Jones et al. (2012) found elevated symptom levels of ASD in birth-assigned females diagnosed with GD but not in birth-assigned males diagnosed with GD. They hypothesized that elevated levels of fetal testosterone may lead not only to reduced empathy, reduced social interest, reduced social skills, and more ASD symptoms (Knickmeyer, Baron-Cohen, Raggatt, & Taylor, 2005; Knickmeyer et al., 2006), but, as part of neurodevelopmental masculinization, also to have a higher chance to develop GD (Jones et al., 2012). Our finding of significantly higher prevalence of the gender item in assigned girls with ASD compared to assigned boys with ASD in adolescents but not in adults only partly supports this hypothesis. As the wish to be of the opposite gender in assigned males at birth remains unexplained by this theory, more research is necessary. A study by Bejerot and Eriksson (2014) in adults with ASD found that tomboyism and bisexuality were overrepresented in females with ASD, but certain other aspects considered as typically masculine (e.g., assertiveness and leadership) were reported to be weaker in both females and males with ASD compared to typically developing controls, suggesting that an extreme male pattern might not apply to all aspects of gender roles and sexuality. A brain MRI study in individuals with ASD also found attenuated typical gender differences in white matter tracts (Beacher et al., 2011), providing support for gender atypicality as one of the potential underlying mechanisms for co-occurring GD–ASD.

Also, contrary to earlier years, there is recent evidence suggesting that more birth-assigned adolescent girls present to specialized gender identity clinics than birth-assigned adolescent boys. This could thus explain the higher odds of the gender item in adolescent girls with ASD compared to adolescent boys with ASD as the ASD sample in the current study had been collected more recently than the sample of the general population that was used as comparison (Aitken et al., 2015; Verhulst et al., 1997). The higher prevalence of endorsement of Item 110 in adolescent girls with ASD compared to adolescent boys with ASD might thus reflect a more broader societal phenomenon instead of suggesting an underlying hypothesis involving testosterone.

Another explanation for co-occurring GD and ASD may be the tendency of individuals with ASD for an intense focus on or an obsessional interest in specific activities, as was suggested by VanderLaan et al. (2014). VanderLaan et al. suggested that when children with ASD form such intense interests in cross-gender objects or activities, a cross-gender identity might develop over time. While typically developing children become more flexible in their stereotyped ideas about gender when they get older (Ruble et al., 2007), children with ASD may be prone to develop GD because of their rigid thinking and difficulty with change (APA, 2013). However, if this were true, the stereotyped subdomain, including repetitive behavior and difficulty with change C/ASBQ subdomain should particularly be elevated in our participants, but the present data did not fully support this idea. This is in line with reports of some clinicians working with GD who do not report obsessional interests as a drive to cross-gender identification (Strang et al., 2018).

According to our third hypothesis, in adolescents there was no significant association between endorsement of Item 110 and any of the specific subdomains of ASD as measured by the CSBQ. In adults, there were only small correlations, but not specifically with regard to limited, repetitive, stereotyped patterns as measured by the ASBQ. Especially in adults, it might be that other ASD characteristics are also involved in the potential co-occurrence between ASD and GD that deserve further study. For example, their specific neuropsychological profiles with deficits in “theory of mind,” the ability to attribute mental states (beliefs, intents, desires, etc.) to oneself and others and recognize that these are different from one’s own, may hamper development of the “self” in general (Lombardo & Baron-Cohen, 2010) and gender identity development more specifically.

According to our fourth hypothesis, of clinical relevance is the finding of our study that emotional problems were more prevalent in adolescents and adults with ASD and co-occurring endorsement of Item 110 compared to those without, especially internalizing problems (depression, anxiety, and somatic complaints). We also found increased problems on all other subscales of the YSR/ASR among those who endorsed sometimes or often wanting to be the opposite gender, but only the internalizing problems were in the clinical range. This is in contrast with the findings of Strang et al. (2014) who did not find increased emotional problems and argued that due to reduced awareness of gender and gender nonconformity among individuals with ASD with increased endorsement of Item 110, they might experience less stigma. Still, the current literature on gender nonconformity gives ample evidence for reduced psychological well-being mainly mediated by experienced stigma (e.g., Baams, Beek, Hille, Zevenbergen, & Bos, 2013), experienced victimization, and ostracism (e.g., Toomey, Ryan, Diaz, Card, & Russell, 2010). Children and adolescents with GD show more self-destructive behavior, including self-harm and suicide attempts (Scourfield, Roen, & McDermott, 2008). As we do know that individuals with ASD have social deficits which put them at risk for a sense that they are “different” from others (de Vries et al., 2010), and with the addition of GD-related stigma, they might be extra prone to experience stigma and show more emotional problems. Further, it might be even more difficult for them than for people without ASD to be open about their gender-variant feelings and “come out,” a step that is thought to be helpful in improving mental health of gender-variant individuals (e.g., Kuyper & Fokkema, 2011). Therefore, clinicians caring for individuals with ASD should realize that their clients might struggle with gender-variant feelings and, if they exist, pay attention to them in an open and nonjudgmental way.

The current findings should be interpreted in light of various limitations. First, the use of only one item of the YSR/ASR (the wish to be of the opposite gender) is a very restricted measure for one aspect of GD. This measure does not acknowledge the non-binary gender identities that are increasingly recognized (Richards et al., 2016). The possibility of responding to this item with “sometimes” or “often” also does not represent the wide variation that is found when these feelings occur. This wish is often not an easily classified “yes” or “no” answer, but a gradual continuum on several aspects of oneself; psychological and physical, ambivalent or incongruent, and with or without a wish for medical gender confirming interventions (see Kuyper & Wijsen, 2014). While the question “wishes to be of the opposite gender” might be sufficient to explore this wish in some individuals with ASD, the rigidity, problems with abstract thinking, and “black and white thinking” might further bias the answering of these questions (e.g., because certain privileges are seen as part of the opposite gender). Therefore, in future studies, it is advisable to use another measure and also qualitative study techniques to examine symptoms of GD.

Second, this study did not make use of a control group other than the YSR/ASR standardization samples. For the YSR, this was a Dutch sample (Verhulst et al., 1997), making comparisons possible, but for the adult sample this was a U.S. sample (Achenbach & Rescorla, 2003). At present, there are, to our knowledge, no U.S. studies on the wish to be of the opposite gender that suggest the prevalence in North America is very different compared to the Netherlands. At the moment, there is some limited evidence that children and adolescents with GD and gender incongruence experience less social ostracism in the Netherlands compared to Canada (Steensma et al., 2014). If this is also true for adults (with or without the wish to be of the opposite gender), it might be easier to report these feelings. The prevalence of reported endorsement of the gender item in our adults with ASD could therefore be somewhat lower if compared with a Dutch adult standardization sample. If increased social ostracism occurs in the U.S., it is expected that expressing feelings of GD is more demanding compared to the Netherlands. Therefore, our finding that individuals with ASD more often experience the wish to be of the opposite gender compared to the standardization sample might be a “sociological” finding of the Dutch population in general experiencing more of those feelings. However, two recent studies on transgender prevalence estimates, with 0.2–0.6% reporting gender dysphoric feelings in a Dutch population study (Kuyper & Wijsen, 2014) and 0.6% in a recent U.S. population study (Flores, Herman, Gates, & Brown, 2016), are making a social explanation only for our results less likely.

In addition, unlike the Strang et al. (2014) study, we did not use other referred control groups. While in their study an overrepresentation of endorsement of Item 110 was found not only in children with ASD, but also in those with ADHD, it would be important to include other referred controls in future studies on the potential occurrence of feelings of GD. It might be that the wish to be of the opposite gender, compared to the general population, is not only overrepresented in individuals with ASD, but also in people with other conditions as well, which might call for different explanations.

Finally, although the use of self-report is a common methodology, the reliability of this method might be different in individuals with ASD compared to typically developing individuals. Individuals with ASD might have problems with introspective ability because of problems with theory of mind, and preliminary evidence suggests caution in the use of self-report measures in adolescents with ASD (Mazefsky, Kao, & Oswald, 2011).

In sum, the present study further confirmed a possible association between ASD and the wish to be of the opposite gender by establishing increased endorsement of this wish in adolescents and adults with ASD compared to the general population controls. Strang et al. (2018) provided a first clinical guideline for adolescents with feelings of GD and ASD based on expert opinions and mention that clinicians working with individuals referred for GD as well as for ASD should pay attention to this possible co-occurrence. Although the wish to be of the opposite gender is not the same as a clinical diagnosis of GD, this is relevant for clinical practice because clinicians working with clients with ASD should be aware of the possible co-occurrence when a wish to be of the opposite gender is expressed or endorsed in self-report measures. Future studies should focus on the underlying mechanisms of this potential co-occurrence and thereby take into account the neuropsychological profiles of individuals with ASD.

## Footnotes

1 In this article, we will use the term boy/male assigned at birth and girl/female assigned at birth when assigned gender at birth is male or female, respectively, which may be incongruent from the experienced gender in individuals with GD.

2 From now on, “the wish to be of the opposite gender” will be used as the current terminology in this article.
